# Neuroprotective Potential of Seed Extracts: Review of In Vitro and In Vivo Studies

**DOI:** 10.3390/nu15112502

**Published:** 2023-05-27

**Authors:** Gabriella Mendes Duarte, Francisco Emanoel Alves de Araújo, João Matheus Caé da Rocha, Francisca Idalina Neta, Amália Cinthia Meneses do Rego, Irami Araújo Filho, Francisco Irochima Pinheiro, Eduardo Pereira de Azevedo, Ricardo Ney Cobucci, Fausto Pierdoná Guzen

**Affiliations:** 1 Postgraduate Program in Biotechnology, Health School, Potiguar University (UnP), Natal 59056-000, Brazil; 2 Graduating in Pharmacy, Health School, Potiguar University (UnP), Mossoró 59056-000, Brazil; 3 Graduated in Nutrition, Health School, Potiguar University (UnP), Mossoró 59056-000, Brazil; 4 Postgraduate Program in Physiological Sciences, Department of Biomedical Sciences, Faculty of Health Sciences, State University of Rio Grande do Norte (UERN), Mossoró 59610-210, Brazil; 5 Laboratory of Experimental Neurology, Department of Biomedical Sciences, Faculty of Health Sciences, State University of Rio Grande do Norte (UERN), Mossoró 59610-210, Brazil; 6 Medical School, Health School, Potiguar University (UnP), Natal 59056-000, Brazil; 7 Postgraduate Program in Health Sciences, Federal University of Rio Grande do Norte, Natal 59010-180, Brazil; 8 Department of Surgical, Federal University of Rio Grande do Norte, Natal 59010-180, Brazil; 9 Graduate Program in Science Applied to Women’s Health, Medical School, Federal University of Rio Grande do Norte, Natal 59010-180, Brazil; 10 Postgraduate Program in Health and Society, Department of Biomedical Sciences, Faculty of Health Sciences, State University of Rio Grande do Norte (UERN), Mossoró 59610-110, Brazil

**Keywords:** seeds, neurodegenerative diseases, neuroprotection, experimental models

## Abstract

Introduction: Neurodegenerative diseases are characterized by neuronal dysfunction and death. Studies suggest that some seed extracts have a neuroprotective effect. Considering the increased incidence of these diseases and the need for new effective therapies with fewer side effects, this review aimed to assess the evidence of the efficacy and safety of seed extracts in experimental models of neurodegeneration. Material and Method: The search was carried out through studies published between 2000 and 2021 in Science Direct, PubMed, Scientific Electronic Library Online (SciELO), and Latin American Literature in Health Sciences (LILACS) databases, in which the effects of seed extracts in in vitro and in vivo experimental models of neurodegeneration were investigated. Based on the eligibility criteria, 47 studies were selected for this review. Results: In the in vitro models, the neuroprotection of the seed extracts was a result of their antioxidant, anti-inflammatory, and anti-apoptotic properties. In the in vivo models, neuroprotection resulted from the antioxidant and anti-inflammatory properties, a decrease in motor deficits, an improvement in learning and memory, as well as the increased release of neurotransmitters. The results show promise for the future of clinical research on new therapies for neurodegenerative diseases. However, the studies are still limited, which does not allow us to extrapolate the results to human beings with ND. Conclusions: Therefore, clinical trials are needed in order to prove the results of the in vitro and in vivo studies, as well as to assess the ideal, safe, and effective dose of these seed extracts in patients with neurodegenerative diseases.

## 1. Introduction

Neurodegenerative diseases are among the causes of the growing mortality and morbidity worldwide, especially in the elderly population. Neurodegenerative disorders are heterogeneous in their clinical presentations and underlying physiology, although they often have overlapping features [1,2,3]. They usually consist of disorders caused by neurodegeneration with subsequent neuronal death. These changes in the nervous system can lead to a decline in the patient’s cognitive functions, and although the causes remain unknown, some are known to be caused by genetic mutations or are due to lifestyle [4].

Neurodegenerative diseases present some challenges regarding their mapping. Alzheimer’s disease is the most common and prevalent dementia worldwide, accounting for about 60–80% of all dementia cases and affecting 24 million people worldwide [5,6,7]. Due to the aging of the population, Alzheimer’s disease is expected to more than triple in 2050 [8]. Parkinson’s disease is a prevalent neurodegenerative disease that is age-related, as is Alzheimer’s disease. A meta-analysis reported an increase from 1% to 4% in the prevalence of Parkinson’s disease in American men aged from 45 to 54 years old, whereas in women in the same age group, it increased from 1% to 2% [9]. Evidence points out that the prevalence of Parkinson’s disease is expected to continue to increase, doubling over the next two decades [9,10].

Although the pathophysiology of these diseases still does not present definitive pathways, it is well known that specific proteins such as beta-amyloid and alpha synuclein are involved in triggering inflammatory and neurodegenerative processes, in addition to genetic and epigenetic changes [11]. However, even though they are typically defined by specific protein accumulations and anatomical vulnerability, neurodegenerative diseases share many fundamental processes associated with progressive neuronal dysfunction and death, such as proteotoxic stress and its concomitant abnormalities in the ubiquitin/proteasomal and autophagosomal/lysosomal systems, as well as oxidative stress, programmed cell death, and neuroinflammation [12].

Treatments for these diseases are aim to relieve the symptoms. As neurodegeneration progresses, the efficacy of pharmacological treatments is diminished, even with increased drug concentrations. Therefore, there is a need to search for more effective treatments for these diseases [13]. Considering the ever-increasing incidence of neurodegenerative diseases and the associated difficulty in finding new effective therapies, the potential preventive and interventional effects of phytochemicals should not be ruled out.

Several seed extracts have shown neuroprotective effects in in vitro models of Alzheimer’s disease [14,15] and in small rodent models by improving memory and learning [16], as well as improving cognitive functions [17,18]. In Parkinson’s disease, neuroprotective effects were also observed in animal models [19]. In addition, seed extracts have been shown to attenuate inflammation in in vitro [20] and in vivo [21] models, as well as to reduce reactive oxygen species in cell models [22]. Thus, extracts from natural products show some advantages, as their natural compounds have several simultaneous targeting approaches that can make them a potential treatment option for Alzheimer’s and Parkinson’s diseases [23]. Thus, several drugs from natural sources have been widely used as therapeutic resources in neurodegeneration [24,25].

Therefore, this review aimed to synthesize the scientific evidence on the neuroprotective effects of plant seed extracts in in vivo and in vitro neurodegeneration models.

## 2. Methodology

This is a narrative review of the literature on studies published between 2000 and 2022 in ScienceDirect, PubMed, Scientific Electronic Library Online (SciELO), and Latin American Literature in Health Sciences (LILACS) databases, in which the effects of seed extracts in experimental models of neurodegeneration were investigated. The search strategy was based on the combination of terms from the Medical Subject Headings (MeSH) such as “Entry Terms”: “Seeds”, “Neurodegenerative Diseases”, “In Vitro”, “Experimental Animal Models”, and “Neuroprotection”. In order to further filter the studies in the databases, the Boolean operators “and” or “or” were used as a search strategy.

The studies included in this review investigated the neuroprotection effect of seed extracts on memory, cognition, neurotrophic factors, inflammatory markers, and oxidative stress biomarkers by using in vitro and in vivo experimental models of neurodegeneration. Studies were excluded if they met one of the following criteria: (1) review studies; (2) studies not peer-reviewed (such as guidelines and preprint); (3) studies performed with seed extracts associated with other herbal supplements, medications, or therapy; (4) double publication (if the article appeared more than once in one of the databases, only the original manuscript was included); (5) experimental models other than small rodents (rats and mice) or cell culture; (6) study carried out with extracts from more than one seed or from another part of the plant other than the seed, such as the stem, leaf, fruit, root, and flower.

## 3. Results

The search strategy used in this review resulted in 883 potentially eligible articles, of which 189 were duplicates and were excluded. The authors independently reviewed each title and abstract for their inclusion. Of the 694 that remained, 617 were excluded after reading the title and abstract, and 77 articles met our selection criteria. However, after reading the articles in their entirety by two authors, 47 original studies remained. No relevant article was identified based on each article’s references.

The studies included are displayed in Table 1 and are organized by authorship, year of publication, anatomical/cellular structure analyzed, experimental model of neurodegeneration, seed categories, as well as their respective extracts and their main results.

### 3.1. In Vitro Models

#### 3.1.1. *Cassia obtusifolia* L.

Ju et al. [32] investigated the effects of ethanolic extracts of *Cassia obtusifolia* L. seeds in a model of Parkinson’s disease, whose neurotoxicity was induced by 6-hydroxydopamine in rat mesencephalic Pheochromocytoma-12 cells. Outcomes were significant and showed the attenuation of cell damage by preventing cell loss from 6-hydroxydopamine stress. Furthermore, the extracts were able to inhibit the overproduction of reactive oxygen species, showing free radical scavenging activity in the 2,2-diphenyl-2-picrylhydrazyl and 2,7-dichlorodihydrofluorescein diacetate assays.

#### 3.1.2. *Alpinia katsumadai*

In an ethanolic extract of *Alpinia katsumadai* seeds, a flavonone named (2*R*, 3*S*)-pinobanksin-3-cinnamate, together with other compounds, showed neuroprotective effects on Pheochromocytoma-12 cells damaged by corticosterone, which may be associated with the elimination of intracellular reactive oxygen species [22].

#### 3.1.3. *Glycine max* L.

The neuroprotective effect of anthocyanins extracted from black soybean, bean seed coat Glycine max were evaluated in a model of ischemia induced by oxygen-glucose deprivation and glutamate-induced cell death in primary cortical neurons of rats for seven days. Anthocyanins showed an antioxidant effect, managing to decrease excessive reactive oxygen species production and to preserve the mitochondrial membrane potential in primary neurons exposed to oxygen-glucose deprivation, but anthocyanins failed to attenuate glutamate-induced excitotoxicity, thus not protecting neuronal cell death induced by glutamate [35].

#### 3.1.4. *Perilla frutescens* L.

A model of increase in reactive oxygen species/cytotoxicity by hydrogen peroxide in primary cultured cortical neurons and in cultured human neuroblastoma cells was performed. The seed extracts obtained from luteolin were used in this experimental model, in which the neuroprotective effects for neural cells insulated by reactive oxygen species were observed. Luteolin isolated from the seeds was able to reverse hydrogen peroxide-induced cytotoxicity in primary cultured cortical neurons; however, in cultured human neuroblastoma cells, such an outcome was not obtained. Furthermore, the effects of luteolin on primary cortical neurons were able to increase their survival. Thus, luteolin extracts isolated from *Perilla frutescens* L. seeds were able to attenuate the increase in reactive oxygen species production and to prevent a significant decrease in the activity of mitochondria, catalase, and glutathione in neurons [36].

#### 3.1.5. *Litchi chinensis*

The effects of *Litchi chinensis* seed extracts were evaluated using a neuronal apoptotic model induced by β amyloid 25–35 by regulating apoptotic pathways and nuclear factor kappa B in Pheochromocytoma-12 cells. Pheochromocytoma-12 cell exposure was 20 μmol/L β amyloid 25–35 for 12 h. The saponins isolated from the *Litchi chinensis* seed extracts were able to significantly inhibit the apoptosis as a result of β amyloid administration 25–35 by the mechanisms of regulation of the apoptotic and nuclear factor kappa B pathways. The mechanisms of inhibition of apoptosis induced by β amyloid 25–35 were associated with the improvement of mitochondrial function, the upregulation of Bcl-2 protein expression, the downregulation of Bax protein expression, the nuclear translocation of NF -κBp6, and the decrease in the expression of messenger RNA [49]. In a recent study, lychee seed polyphenol was evaluated in an Alzheimer’s disease model using a culture of BV-2 cells and Pheochromocytoma-12 cells induced by β amyloid. *Litchi chinensis* extracts were able to inhibit neuroinflammation mediated by the β amyloid (1–42)-induced Nod-like receptor family pyrin domain containing 3 inflammasome activation. The mechanism was associated with the inhibition of the Nod-like receptor family pyrin domain containing 3 expression, caspase-1 cleavage, and interleukin--1β release in β amyloid-induced BV-2 cells (1–42). In addition, polyphenols from the *Litchi chinensis* seed extracts improved Pheochromocytoma-12 cell viability, therefore decreasing cell death [60].

#### 3.1.6. *Cannabis sativa* L.

Several compounds have been isolated from hemp seed extracts (*Cannabis sativa*). In one study, cannabisin F was isolated for the evaluation of its neuroprotective effect in a model of inflammation and oxidative stress induced by lipopolysaccharides in BV2 microglial cells. The results showed the significant attenuation of inflammatory responses and a decrease in the production of reactive oxygen species associated with the expression pathway of the enzyme sirtuin 1/nuclear factor kappa B and nuclear factor 2 related to erythroid-2. The anti-inflammatory activity was responsible for suppressing the production of pro-inflammatory mediators, such as interleukin 6 and tumor necrosis factor α, which was associated with the modulation expression of enzyme sirtuin 1 and the suppression of the activation of the nuclear factor kappa B signaling pathway. Furthermore, the antioxidant activity of *Cannabis sativa* seed extract was involved in the reduction of reactive oxygen species and in the expression of nuclear factor 2 related to erythroid-2 and heme oxygenase-1 (HO-1) [20]. In another study, the anti-inflammatory and antioxidant effects of nuclear factor kappa B and nuclear factor 2 related to erythroid-2 pathways were also observed in a model of lipopolysaccharide-induced neuroinflammation in BV2 microglia, using the isolated glucopyranoside phenylpropionamide, coumaroylaminobutanol from *Cannabis sativa* seed. Coumaroylaminobutanol glucopyranoside was able to significantly decrease proinflammatory cytokine and adenosine monophosphate-activated protein kinase expression, as well as to induce the suppression of the nuclear factor kappa B signaling pathway by inhibiting the phosphorylation of the nuclear factor kappa B inhibitor and nuclear factor kappa B p65. In addition, coumaroylaminobutanol glucopyranoside was able to significantly reduce the production of reactive oxygen species [54].

#### 3.1.7. *Camellia sinensis*

A model of hydrogen peroxide-induced cytotoxicity in Pheochromocytoma-12 cells with the characteristics of adrenal gland blastoma cell lines was performed. In this study, green tea seed oil was used to assess its neuroprotective effect on Pheochromocytoma-12 cells. The study concluded that green tea seed oil increased cell viability and significantly reduced reactive oxygen species production in Pheochromocytoma-12 cells with hydrogen peroxide-induced cytotoxicity [18]. In the study conducted by Khan et al., the effects of saponin E1 isolated from *Camellia sinensis* seeds in an Alzheimer’s disease model with mouse neuroblastoma cells (SweAPP N2a) were evaluated. A significant reduction of β amyloid was observed in the treated cells, showing a reduction in its production by inhibiting the amyloidogenic cleavage of amyloid precursor protein. Thus, a significant reduction in β amyloid formation and accumulation of the activation of α-secretase and neprilysin was observed. In addition, a significant reduction in acetylcholinesterase activity was observed [62].

#### 3.1.8. *Hibiscus sabdariffa*

A study was carried out by Shalgum et al., in which the neuroprotective effects of extracts from *Hibiscus sabdariffa* seeds in a model of neurotoxicity induced by the endogenous hydrogen peroxide neurotoxin in cell line SH-SY5Y were evaluated. Treatment with the seed extract of *Hibiscus sabdariffa* showed significant antioxidant and anti-apoptotic effects, managing to attenuate the neurotoxicity induced by hydrogen peroxide [55].

#### 3.1.9. *Reynoutria elliptica*

Song et al. [56] evaluated the neuroprotective effects of procyanidin isolated from *Reynoutria elliptica* seeds in a glutamate-induced cell death model in the murine hippocampal HT22 cell line. The seed extracts showed a significant antioxidant response as a result of glutamate-induced cell death. Such activity was able to block the glutamate-mediated increase in intracellular reactive oxygen species accumulation and the phosphorylation of mitogen-activated protein kinase, resulting in the prevention of glutamate-induced apoptotic cell death. Furthermore, it was able to significantly inhibit the phosphorylation of the extracellular signal-regulated kinase, p38, and N-terminal c-Jun kinase, which had increased by glutamate induction.

#### 3.1.10. *Vitis vinifera* L.

In previous studies with primary cultures of astrocytes in the hippocampus in a model of oxidative stress induced by hydrogen peroxide, grape seed extract was able to increase the production of interleukin-6, which managed to protect neuronal cell death induced by oxidative stress [30]. In more recent models of ethanol-induced hippocampal neuronal injury and oxidative stress in primary cultures of rat hippocampal neurons, treatment with grape seed procyanidin extract was performed with the purpose of evaluating their neuroprotective property. The results showed that grape seed procyanidin was able to attenuate ethanol-induced neuronal toxicity. Grape seed procyanidin was able to avoid the neuronal damage induced by ethanol, significantly decreasing the levels of malondialdehyde and lactate dehydrogenase while increasing the activity of superoxide dismutase. Furthermore, repair was observed in the neuronal morphology previously damaged by ethanol, increasing the amounts of dendrites and the total dendritic length per cell [59]. The effects of grape seed procyanidin extract were investigated using a hydrogen peroxide-induced oxidative stress neurodegeneration model in Pheochromocytoma-12 cell neuroblastoma. Significant results have been observed in the attenuation of oxidative stress, which was influenced by the degree of polymerization and the structural characteristics of grape seed procyanidin extract [58]. He et al. [63] evaluated the neuroprotection activity of grape seed procyanidin extract in a model of damage induced by hydrogen peroxide inPheochromocytoma-12 cells. The results of the study showed that grape seed procyanidin extract was able to significantly reduce the levels of intracellular reactive oxygen species and apoptosis induced by hydrogen peroxide. Such effects were associated with the phosphoinositide-3-kinase–protein kinase B/Akt signaling pathway. Grape seed procyanidin extracts have also been studied in cellular models of oxygen-glucose deprivation/reoxygenation ischemic injury. In addition, mouse neuroblastoma N2a cells were used in this study, and grape seed procyanidin extract was able to increase cell viability and inhibit apoptosis after ischemic injury [53].

#### 3.1.11. *Celosia argentea* L.

In the study conducted by Guo et al. [64] a model of neuronal injury induced by tert-butyl hydro-peroxide in an NSC-34 cell culture was performed. In this model, the NSC-34 cell culture was treated with triterpenoid saponins and phenylacetonitrile glycosides isolated from *Celosia argentea* L seeds. These compounds showed marked antioxidant responses, significantly attenuating the neuronal damage induced by tert-butyl hydro-peroxide mainly by decreasing reactive oxygen species generation. The proposed mechanism of its antioxidant effects may be associated with the upregulation of the expression and activation of some antioxidant enzymes in the autophagy pathway.

#### 3.1.12. *Mucuna pruriens* (L.)

Rachsee et al. [65] investigated the anti-inflammatory activity of *Mucuna pruriens* seed extracts on BV2 mouse microglial cells by lipopolysaccharide-stimulated inflammatory responses. The study showed that the seed extract of *Mucuna pruriens* was able to significantly reduce inflammatory responses by inhibiting the nuclear factor kappa B signaling pathway, thus decreasing the production and release of pro-inflammatory mediators in lipopolysaccharide-stimulated BV2 cells. Thus, it was observed that the release of inflammatory mediators, including nitric oxide, interleukin-1β, interleukin-6, and tumor necrosis factor α, were significantly inhibited by the seed extract of *Mucuna pruriens*.

#### 3.1.13. *Celastrus paniculatus*

The water-soluble extracts of *Celastrus paniculatus* seeds were evaluated in a model of oxidative damage induced by hydrogen peroxide in cultures of rat forebrain neuronal cells. The seed extracts were able to reduce the levels of free radicals, decrease lipid peroxidation and induce the enzyme catalase, thus protecting against neuronal damage and significantly attenuating neuronal death [26]. In addition, it has been reported that the seed extract of *Celastrus paniculatus* protects against glutamate-induced toxicity in rat forebrain neuronal cultures. The administration of *Celastrus paniculatus* seed extracts protected the neuronal cells against glutamate-induced toxicity due to the modulation of the glutamatergic receptor [27].

### 3.2. In Vivo Models

#### 3.2.1. *Alpinia katsumadai*

Extracts from *Alpinia katsumadai* seeds were investigated in several neuroprotection models. In the study conducted by Li et al. [34] a model of transient ischemic damage in the hippocampus of gerbils was used to assess the effect of *Alpinia katsumadai* seeds pre-treatment. Gerbils received an oral administration (25 and 50 mg/kg) of *Alpinia katsumadai* seeds once daily for 7 days prior to transient cerebral ischemia. In the group treated with 50 mg/kg of *Alpinia katsumadai* seeds, about 67% of neurons in the CA1 region of the hippocampus survived after ischemia/reperfusion. In addition, brain-derived neurotrophic factor protein levels in the ischemia-treated group with 50 mg/kg of *Alpinia katsumadai* seeds were much higher than those in the ischemia-treated group. Thus, the outcomes showed that *Alpinia katsumadai* seeds managed to protect neurons against ischemic damage in the hippocampus. In another study using a similar model of ischemic damage in the CA1 region of the gerbil hippocampus after transient cerebral ischemia, the oral administration of 25 and 50 mg/kg of *Alpinia katsumadai* seeds was performed. The results showed antioxidant responses in the oxidative stress control pathway. The immunoreactivity of the enzyme sodium hydrogen cotransporter in the vehicle-ischemia group was distinctly altered in the CA1 region after ischemic damage. However, in the *Alpinia katsumadai* seeds-ischemia group, immunoreactivity was not significantly altered in the CA1 region after ischemia/reperfusion [39].

#### 3.2.2. *Cassia obtusifolia* L.

A model of Parkinson’s disease induced by 1-methyl-4-phenyl-1,2,3,6-tetrahydropyridine was performed in mice pretreated with *Cassia obtusifolia* seed extracts. The results indicated that the *Cassia obtusifolia* extract-treated group achieved a marked protection against 1-methyl-4-phenyl-1,2,3,6-tetrahydropyridine-induced neuronal degeneration in the substantia nigra and striatum of mice [32]. In another study, an Alzheimer’s disease model was performed by Yi et al. [44], in which mice were induced to aberrant synaptic plasticity and memory impairment by β amyloid. The hippocampus of mice was evaluated, and *Cassia obtusifolia* extracts were able to generate significant improvement in synaptic dysfunction induced by β amyloid through anti-inflammatory pathways and Akt/GSK-3β, attenuating the activation of microglia induced by β amyloid, inducible nitric oxide synthase, and cyclooxygenase.

#### 3.2.3. *Sesamum indicum* L.

The neuroprotection properties of defatted *Sesamum indicum* seed extract were evaluated in an ischemia-reperfusion injury model by middle cerebral artery occlusion in the brains of rats. The administration of *Sesamum indicum* seed extract after the onset of ischemia reduced the cerebral infarct volume and improved the sensorimotor function of rats induced in the cerebral ischemia model [33]. In a 6-hydroxydopamine-induced neurotoxicity Parkinson’s disease model, *Sesamum indicum* seed extract demonstrated a significant reduction in the activity of antioxidant and non-antioxidant enzymes, such as glutathione reductase, glutathione-S-transferase, glutathione peroxidase, catalase, and glutathione content and thiobarbituric acid reactive substance in the striatum of mice. In addition, significant increases in brain dopamine levels have been shown [37].

#### 3.2.4. *Punica granatum*

A model of cerebral ischemia induced by the bilateral permanent occlusion of the common carotid arteries was performed in albino female rats to evaluate the effects of *Punica granatum* seed extracts. Seventy female Wistar rats were randomly divided into seven groups of ten each. The oral administration of *Punica granatum* seed extracts significantly improved active and passive avoidance memory in female rats, and its ameliorative effect was associated with the antioxidant activity of *Punica granatum* seed extracts and its ability to scavenge free radicals [38].

#### 3.2.5. *Camellia oleífera*

The neuroprotective effect of sapogenins isolated from the defatted seeds of *Camellia oleiferae* was evaluated in an animal model of Parkinson’s disease using mice and induced by 1-methyl-4-phenyl-1,2,3,6-tetrahydropyridine. The results showed that sapogenin and its derivatives increased dopamine levels in the striatum and tyrosine hydroxylase positive cells in the substantia nigra. In addition, inflammation was reduced, and motor deficits were ameliorated. The effect on movement was reversed by the dopamine receptor antagonist haloperidol rather than the free adenosine receptor antagonist [19].

#### 3.2.6. *Nigella sativa*

The seed extract of *Nigella sativa* was evaluated in an experimental model of multiple sclerosis with neuroinflammation and demyelination in Wistar rats induced by autoimmune encephalomyelitis. The *Nigella sativa* seed extract was able to attenuate autoimmune encephalomyelitis-induced neuroinflammation with a significant increase in remyelination in the cerebellum of rats. In addition, it reduced the expression of transforming growth factor beta 1 [41].

#### 3.2.7. *Celastrus paniculatus*

The effects of the ethanolic extract of *Celastrus paniculatus* seeds in a model of neurotoxicity induced by 3-nitropropionic acid in male Wistar rats were evaluated. Animals treated with the extract of *Celastrus paniculatus* (100 and 200 mg/kg) showed an improvement in behavioral parameters and oxidative stress compared to animals treated with 3-nitropropionic acid [45]. Neuroprotective effects were also reported in a model of cognitive impairment induced by chronic stress in rats pre-treated with oil from seed extracts of *Celastrus paniculatus* for 14 days. Stressed rats were evaluated through the radial arm maze and T-maze tests. It was observed that the oil from the seed extracts was able to significantly reduce stress-induced anxiety. In addition, a significant improvement in cognitive deficits and the restoration of spatial learning and memory was reported [42].

#### 3.2.8. *Cicer microphyllum*

The study conducted by Sharma et al. [46] evaluated the effects of ethanolic extracts from *Cicer microphyllum* seeds in improving global neurodegeneration induced by hypoxia in the CA1 region of the hippocampus. Study outcomes showed antioxidant activities resulting in reduced lipid peroxidation, protein oxidation, and DNA damage as a result of exposure to chronic hypoxia. Extracts of *Cicer microphyllum* were able to significantly reduce neurodegeneration and dendritic atrophy in CA1 neurons. Extracts from *Cicer microphyllum* also induced dendritic arborization via estrogen receptor beta activation and extracellular signal-regulated kinase phosphorylation. Thus, extracts from *Cicer microphyllum* proved to be effective and safe in attenuating hypoxia-induced cognitive and memory impairment.

#### 3.2.9. *Phoenix dactylifera* L.

Dehghanian et al. [16] investigated the effects of *Phoenix dactylifera* seed extract in an β amyloid-induced Alzheimer’s disease model in the hippocampus in 24 male rats. The results showed that the administration of *Phoenix dactylifera* seed extract in rats resulted in antioxidant responses, which restored β amyloid-induced memory and learning deficiencies, reduced the expression of caspase-3, and decreased the degenerated neurons in the CA1 subfield of the hippocampus.

#### 3.2.10. *Mucuna pruriens*

Trials to assess the neuroprotective properties of *Mucuna pruriens* have aroused increasing interest about its traditional use in India as a neuroprotective agent. In previous studies in a subchronic mouse model of Parkinson’s disease induced by 1-methyl-4-phenyl-1,2,3,6-tetrahydropyridine, *Mucuna pruriens* seed extract was not able to prevent 1-methyl-4-phenyl-1,2,3,6-tetrahydropyridine-induced tyrosine hydroxylase reduction or microglia activation [31]. However, in more recent studies, such as the one by Yadav et al. [40], a mouse model of Parkinson’s disease induced by chronic exposure to paraquat was performed. The mice were treated with the aqueous extract of *Mucuna pruriens* and a significant reduction in the oxidative stress of the nigrostriatal tissue and improvement in neurobehavioral activity were observed. The results of the study showed a significant reduction in paraquat-induced neurotoxicity. The nigrostriatal portion of the mouse brain presented higher levels of nitrite and malondialdehyde and reduced levels of catalase in comparison to the control group. Furthermore, *Mucuna pruriens* treatment also ameliorates behavioral abnormalities and significantly decreases tyrosine hydroxylase immunoreactivity in the substantia nigra and striatal regions of the mouse brain. In another model of Parkinson’s disease by neuroinflammation induced by 1-methyl-4-phenyl-1,2,3,6-tetrahydropyridine intoxication in mice, the effects of the aqueous extract of *Mucuna pruriens* (100 mg/kg) administered orally were evaluated. *Mucuna pruriens* treatment was able to inhibit lipid peroxidation and decrease nitrite levels. In addition, it improved catalase activity and increased glutathione levels in the nigrostriatal region of the mice’s brain. Increased tyrosine hydroxylase and dopamine transporter immunoreactivity were also observed. Significant relief in 1-methyl-4-phenyl-1,2,3,6-tetrahydropyridine-induced neurotoxicity by the nuclear factor kappa B and pAkt1 pathways was also observed, which resulted in the further prevention of apoptosis of dopaminergic neurons, as well as in the improvement of neuroinflammation and behavioral abnormalities [47].

#### 3.2.11. *Trigonella-foenum graecum* L.

A study conducted by Assad et al. [50] investigated the methanol extract of *Trigonella foenum-graecum* Linn as a neuroprotective agent in mice. An exteroceptive and interoceptive behavioral model of amnesia was induced by scopolamine to assess the process of learning and memory. The animals that received 200 mg/kg of methanol extract of *Trigonella foenum-graecum* Linn exhibited a decline in transfer latency on days of both acquisition and retention. Therefore, significant improvement in the learning and memory process was observed in scopolamine-induced mice treated with the methanol extract of *Trigonella foenum-graecum* Linn.

#### 3.2.12. *Moringa oleífera*

A model of cognitive impairment induced by the injectable administration of scopolamine in mice was performed through two tests: passive avoidance and Morris water maze. A total of 250 or 500 mg/kg of *Moringa oleifera* seed ethanol extract was administered to mice orally for 7 or 14 days. Pretreatment with *Moringa oleifera* seed ethanol extract significantly enhanced cognitive impairment, increased cholinergic system reactivity, and improved neurogenesis in the hippocampus. *Moringa oleifera* seed ethanol extract-enhanced cognitive effects were mainly associated with increased cholinergic neurotransmission and neurogenesis via the activation of Akt and extracellular signal-regulated kinase phosphorylation pathways [52]. In a model of ischemic stroke induced by acute and late-stage brain damage in rats, significant results in cerebrovascular protection were observed after the administration of extract. Its neuroprotection mechanism was associated with hippocampal neurogenesis, synaptic plasticity, and improved cholinergic function [58].

#### 3.2.13. *Litchi chinensis*

In a model of Alzheimer’s disease induced by the injection of β amyloid 25–35 into the lateral ventricle of rats, cognitive functions and apoptosis were evaluated after the administration of *Litchi chinensis* seed extract. The results showed that this extract was able to significantly improve cognitive function and alleviate neuronal damage by inhibiting apoptosis in the hippocampus [17]. In another model of Alzheimer’s disease by neuronal damage induced by the administration of β amyloid 25–35 in the hippocampus of rats, the *Litchi chinensis* seed extract was able to attenuate the lesion and improve the cognitive functions of rats through the AKT/GSK-3β pathway [61].

#### 3.2.14. *Cannabis sativa* L.

A model of memory dysfunction and neuroinflammation was induced by lipopolysaccharide in mice to assess the effects of seed extracts of *Cannabis sativa* containing phenylpropionamides. Phenylpropionamides were able to improve learning and alleviated lipopolysaccharide-induced damage to spatial memory. Furthermore, the levels of interleukin (interleukin--1β and interleukin-6) and tumor necrosis factor α in the mice’s brain were significantly increased in the lipopolysaccharide-induced group. Phenylpropionamides were also able to attenuate lipopolysaccharide-induced hippocampal neuronal damage in mice [21].

#### 3.2.15. *Camellia sinensis*

*Camellia sinensis* was administered to mice in a model of behavioral and cognitive deficit induced by β amyloid 1–42. The administration of *Camellia sinensis* was associated with improved behavioral and cognitive deficits in mice induced by β amyloid 1–42 through the Akt pathway related to β amyloid. In addition, significant ameliorative effects have been reported on the parameters of cognitive dysfunction and neurotoxicity through various physiological activities. Thus, *Camellia sinensis* was able to significantly improve the cognitive and behavioral dysfunction induced by β amyloid 1–42 in mice [18].

#### 3.2.16. *Vitis vinifera* L.

Several reports have shown the broad neuroprotective effects attributed to *Vitis vinifera*, which include protection against ischemic neuronal damage by inhibiting DNA damage in the hippocampus after transient forebrain ischemia [28] and reducing hypoxic-ischemic brain damage in newborn rats, with the attenuation of neurofunctional disorders caused by hypoxia-ischemia, associated the with suppression of lipid peroxidation [29]. More recent studies, such as the research by El-Tarras et al. [43], evaluated the effects of *Vitis vinifera* extract in a model of cadmium chloride neurotoxicity using 40 male albino rats. In the brain, evidence of the degeneration of nerve fibers and cells was observed. Fibrosis events and blood vessel congestion have also been reported. In the group of rats treated with *Vitis vinifera* extract, reduction in histopathological changes in brain tissue was observed, even though some blood vessels still showed evidence of congestion. In another model of ischemia-reperfusion brain injury through transient middle cerebral artery occlusion in mice, the effects of *Vitis vinifera* extract were evaluated. Significant functional improvement was observed within 24 h after middle cerebral artery occlusion in the treated group compared to the normal saline group. Furthermore, the group treated with the extract had a lower cerebral systolic volume. Therefore, the study postulated that *Vitis vinifera* extract was able to attenuate oxidative stress and apoptosis. In addition, it activated the antioxidant enzyme glutathione peroxidase [48]. Anti-apoptotic activity attributed to *Vitis vinifera* extract has also been reported in a murine model of neonatal hypoxic-ischemic brain injury. The study showed improvement in brain damage and neurobehavior in the group pretreated with the extract, in addition to suppressing apoptosis by inhibiting the expression of bax and cleaved caspase-3 [57].

## 4. Discussion

**Figure 1:**
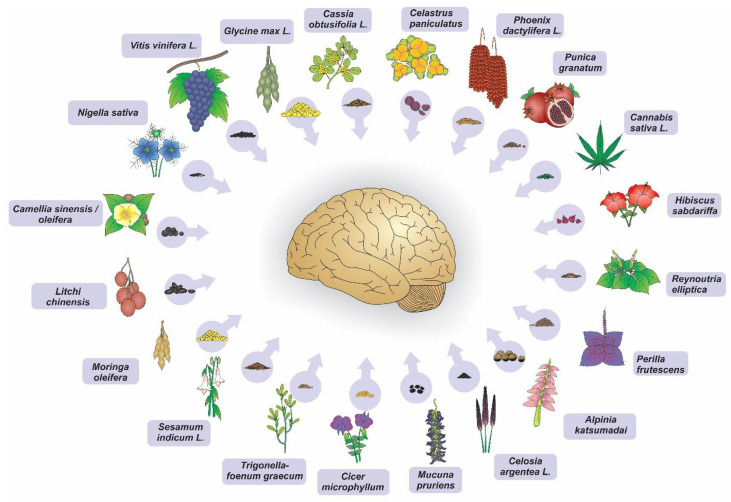
Seed extracts of plants that have shown neuroprotective potential in neurodegeneration models (in vitro and in vivo). (Illustration by Dr. Francisco Irochima).

The majority of the studies included in this review confirmed that there is enough evidence to support the neuroprotective effect of the investigated seeds, as shown in the in vitro and in vivo models (Figure 1).

**Figure 2:**
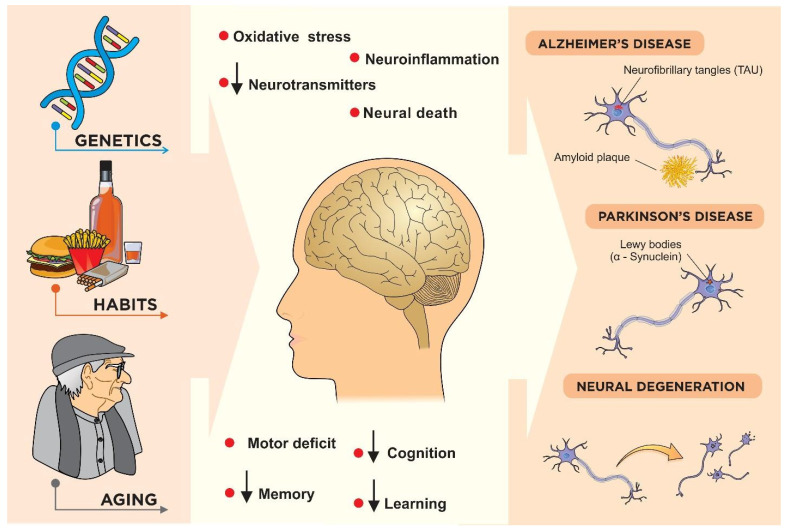
Factors related to neurodegenerative diseases. Genetic aspects and others related to lifestyle and aging may result in oxidative stress, neuroinflammation, decreased levels of neurotransmitters, neuronal death, and motor deficit, as well as decreased memory, cognition, and learning. These deleterious events can cause abnormal accumulations of beta amyloid and TAU proteins, such as those that occur in Alzheimer’s disease, as well as in alpha-synuclein protein, such as those that occur in Parkinson’s disease. (Illustration by Dr. Francisco Irochima).

In in vitro models, neuroprotection occurred mainly through the inhibition of reactive oxygen species production, the prevention of reduction of antioxidant enzymes, a decrease in inflammatory markers, and by anti-apoptotic activity. In the in vivo models, in addition to the elimination of free radicals and the reduction in the inflammatory response, a reduction in motor deficits, improvement in learning and memory, and increase in the release of neurotransmitters, such as dopamine and acetylcholine, were observed. Antioxidants that are present in neuronal tissue are potential candidates for the prevention and treatment of disorders involving oxidative damage [66]. In cell models, antioxidant activity was observed in the groups treated with seed extracts (Figure 2).

Previous chemical studies with *Reynoutria elliptica* have shown the presence of several compounds, including quinones, stilbenes, flavonoids, coumarins, lignans [67], and procyanidins [68]. Among these compounds, procyanidins stand out due to several of their biological properties, including the antioxidant [69], anti-inflammatory [70], and neuroprotective properties [71]. Grape seeds (*Vittis vinifera*) are also known to have procyanidins as their major compounds, which may have influenced the positive neuroprotective results found in the studies covered in this review [66].

The antioxidant activity of naturally occurring triterpenoids has been investigated in several studies [72]. Plants of the Hibiscus family contain numerous neuroprotective compounds, including triterpenoids [73]. Hemp roots are also known to contain considerable amounts of pentacyclic triterpenoids [74]. The saponins present in *Celosia argentea* [75] and *Hibiscus Sabdariffaffa* have remarkable properties, which include antioxidant and anti-inflammatory activities [76]. It is important to highlight that oxidative stress is one of the most critical factors for neuronal cell death, which is characterized by an imbalance between reactive oxygen species production and the cellular defense system. Therefore, preventing oxidative stress is valuable for protecting neurons against damage and for preventing apoptosis [77]. The aforementioned compounds may have contributed to both the antioxidant activity and the reduction of cell death that have been attributed to these seeds.

In vitro studies with *Cannabis sativa* and *Mucuna pruriens* evidenced the attenuation of inflammatory responses. The benefits of hemp seeds (*Cannabis sativa* L.) seem to be attributed to their high contents of oils and proteins. A previous study isolated coumaroylaminobutanol glucopyranoside from *Cannabis sativa* seed, which is a phenylpropionamide compound that presented marked activity in the anti-neuroinflammatory screening test, performed through a lipopolysaccharide-induced BV2 microglia model [78]. The anti-neuroinflammatory activity of *Mucuna pruriens* may be due to the presence of some bioactive constituents such as tannins, alkaloids, phenolic compounds, and flavonoids [47]. Furthermore, natural compounds containing flavonoids and phytochemicals with antioxidant and anti-inflammatory activities have shown neuroprotective effects in neurodegenerative diseases [79].

In the in vivo models, the administration of seed extracts resulted in a significant decrease in the production of intracellular reactive oxygen species and an increase in antioxidant enzymes. In fact, most natural antioxidants are derived from plant sources such as the seeds. Their antioxidant activity is mainly due to the phenolic compounds, tocopherols, carotenoids, and flavonoids, which were present in most of the seeds evaluated in the studies included in this review [80].

The use of in vivo models of neurodegeneration to investigate the neuroprotective activity of seed extracts has some advantages over the in vitro models. The in vivo models enable a better investigation of the neuronal and cognitive functions, as well as inflammation and immune responses. In trials in which ischemia models were used, some seeds demonstrated the ability to reduce cognitive impairment in the animals [34,46]. Sun et al. [61] used an in vivo Alzheimer’s model to report that *Litchi chinensis* seed extract had the potential to improve cognitive function. However, these studies used animal models that were developed in the laboratory for experimental purposes, which probably resulted in different levels of neurodegeneration that might differ from what occurs in humans with ischemia and Alzheimer’s disease.

However, there are some similarities regarding biochemical changes that take place in both in vitro and in vivo models, which normally occur in neurodegenerative diseases that are common in humans, such as ischemic stroke, Parkinson’s disease, and Alzheimer’s disease. Such similarities come from the fact that biochemical changes, such as the proliferation of beta-amyloid proteins, TAU, and alpha-synuclein, may trigger similar inflammatory and neurodegenerative processes in both models [11]. In addition, models of Alzheimer’s and Parkinson’s diseases showed another similarity regarding what occurs in humans affected by these diseases, which is neuronal death that can result in a significant decline in cognitive and motor functions [4].

On the other hand, there are differences in the pharmacokinetics and pharmacodynamics between in vivo models and humans, which might lead to a different response when the extracts are tested in the laboratory in comparison to administration to people with neurodegenerative diseases.

The inflammatory process is closely related to multiple neurodegenerative pathways [81]. In vivo models using *Cassia obtusifolia* and *Camellia oleifera* showed a reduction in inflammatory response. *Cassia obtusifolia* L has many functional compounds, including obtusifolin, alaternin, and rubrofusarin. Furthermore, saponin Sasanqua is a safe compound present in *Camellia oleifera*. Sapogenin is present as well, with many pharmacological activities such as anti-oxidative, anti-inflammatory, immunomodulation, and antitumor properties, which may also play important roles in neuroprotection [82].

The main pharmacological approaches for the treatment of Alzheimer’s disease are based on reducing β-amyloid levels in the brain; however, no significant results have been shown yet, as the outcomes of the available pharmacological treatments rely on the relief of clinical symptoms [83]. In fact, acetylcholinesterase inhibitors are the most commonly prescribed medications for Alzheimer’s disease, as they temporarily increase the availability of acetylcholine at cholinergic synapses [84]. In Alzheimer’s disease models with small rodents, several seed extracts showed neuroprotective effects by significantly improving cognitive function, including *Moringa oleifera* seeds. Previous studies indicated that the extract of this seed is rich in hydrocarbons or long-chain polyunsaturated fatty acids and their derivatives [85]. Considering that polyunsaturated fatty acid supplementation increases synaptic plasticity in the hippocampus and, therefore, enhances cognitive function, treatment with the extract of *Moringa oleifera* seeds seems to be a promising alternative for the management of Alzheimer’s disease [86].

In addition, the cholinergic system plays an important role in cognitive function. The death of cholinergic cells by β amyloid toxicity increases the release of cell-bound acetylcholinesterase, and the increase in acetylcholinesterase accelerates the degradation of acetylcholine in the synaptic cleft [87]. It is suggested that the improvement in cognitive deficit in Alzheimer’s disease models is due to the abundance of unsaturated fatty acids and several triterpenoid compounds, such as C2 ganoderic acid. These compounds appear to directly inhibit acetylcholinesterase activity, resulting in increased levels of acetylcholine.

*Mucuna pruriens* seed extracts have shown to attenuate clinical signs and symptoms in Parkinson’s disease models. In the study conducted by Rai et al. [47], the administration of *Mucuna pruriens* seed extracts resulted in the prevention of apoptosis of dopaminergic neurons, the attenuation of neuroinflammation, and the restoration of behavioral abnormalities in mice with induced Parkinson’s disease. The literature shows that an intense loss of dopaminergic neurons in the substantia nigra, in addition to reduced levels of dopamine, are observed in patients with Parkinson’s disease. Furthermore, prolonged neuroinflammation plays an important role during the degeneration of neurons in Parkinson’s disease [88]. *Mucuna pruriens* is rich in bioactive compounds such as tannins, alkaloids, phenolic compounds, and flavonoids [89]. Moreover, high performance liquid chromatography data evidenced the presence of different phytochemicals, such as proanthocyanidin, tannin, gallic acid, quercetin, and phytic acid, in the aqueous extract of *Mucuna pruriens* seeds, whose anti-neuroinflammatory activities may be due to the presence of these active constituents. Its extracts have also demonstrated potential anti-cataleptic and anti-epileptic properties associated with the presence of dopamine and 5-hydroxytryptamine [90].

Protection against the nigrostriatal degeneration of dopaminergic neurons has been associated with the reduction of oxidative stress and neuroinflammation in Parkinson’s disease mouse models. Furthermore, the effects of *Mucuna pruriens* in protecting against Parkinson’s disease have also been associated with the attenuation of neuroinflammation induced by reactive oxygen species [47]. In Parkinson’s disease neuroinflammation, there is an increase in the activation of glial cells as a cellular response [91]. In fact, the inhibition of glial activation by some inhibitors has already been presented in the literature as a therapeutic resource to prevent induced neurotoxicity by 1-methyl-4-phenyl-1,2,3,6-tetrahydropyridine [92]. Thus, treatment with *Mucuna pruriens* seems to suppress inflammatory responses associated with glial cell activation and dopaminergic neuronal loss [47].

Several bioactive compounds from seed extracts have shown promising results in attenuating clinical signs and symptoms in both in vitro and in vivo models of neurodegeneration. However, although the outcomes were promising, it is important to emphasize some limitations, as some results in humans were inconclusive and demonstrated a limited effect of some of these seeds in attenuating the effects of reactive oxygen species. This seems to be due to the fact that, in clinical trials, usually only single compounds are investigated, but the combination of several active compounds in extracts may lead to synergistic effects and, thus, may potentiate neuroprotective effects in patients affected by Alzheimer’s disease and Parkinson’s disease [93,94].

**Figure 3:**
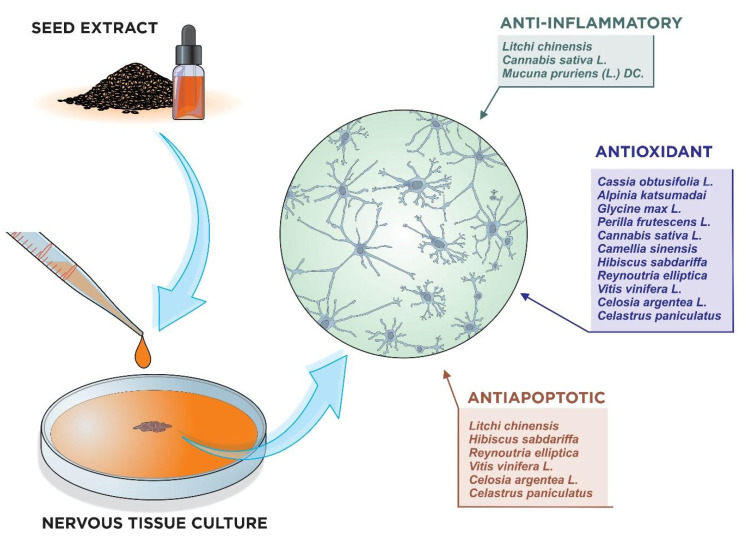
Seeds with anti-inflammatory, antioxidant, and anti-apoptotic properties, as demonstrated by in vitro studies with nerve cell culture. (Illustration by Dr. Francisco Irochima).

Neurodegenerative diseases are associated with multiple cellular dysfunctions as a result of various external and internal factors; therefore, multi-targeted drug therapy seems to be the best approach to ameliorate the symptoms associated with these diseases [95,96]. Although many of these natural compounds are well established as neuroprotective agents in studies with experimental models, many have not been tested in clinical trials; therefore, their use must be avoided in patients with neurodegenerative diseases [97]. That said, although the in vitro models covered in this review revealed that the seed extracts attenuated inflammation and cell death, these results are limited to cells in a controlled environment and their extrapolation to the human organism is not feasible until they are tested in controlled clinical trials (Figure 3).

**Figure 4:**
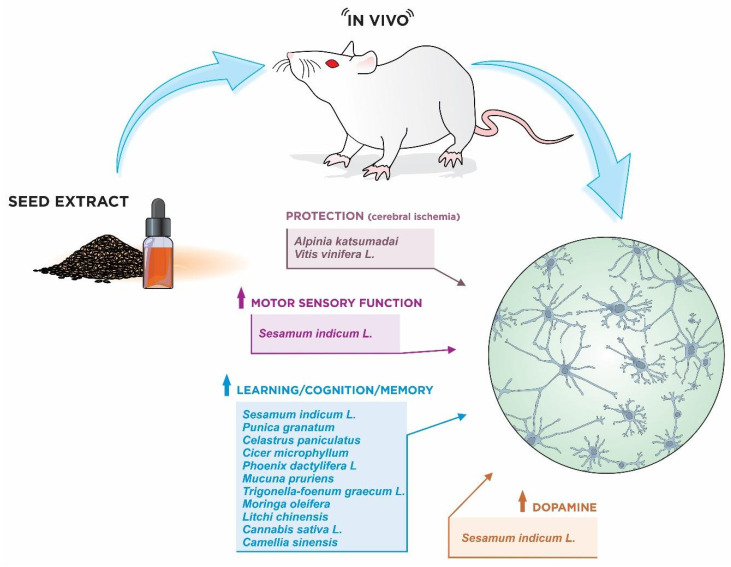
Seeds with protective effects on ischemic brain damage and with improvement in cognitive, learning, memory, and sensorimotor deficits as demonstrated by in vivo animal studies. (Illustration by Dr. Francisco Irochima).

Due to their genetic, anatomical, and physiological similarities, animal models are essential as antecedents to tests in humans. However, despite the similarities, some differences between animals and humans need to be considered in order to better understand the effects of therapies on human biological systems [98]. As an example, the effects on protection against the ischemic neuronal damage of procyanidins from seeds of *Vitis vinifera* L. in rats have some limitations due to anatomical differences compared to human cerebral blood vessels, especially regarding their caliber, which may lead to different results in clinical trials (Figure 4) [48].

Furthermore, a better understanding of the mechanisms that cause neurodegenerative diseases is needed, as in most of these diseases the exact cause is still not fully understood, if not completely obscure. Therefore, ongoing research using animal models is essential to better elucidate these neurological disorders, thus improving and developing new therapeutic alternatives for the treatment of neurodegenerative diseases [99].

## 5. Conclusions

Several compounds naturally occurring in seed extracts have shown promising results in ameliorating clinical signs and symptoms in neurodegeneration models through attenuating the responses resulting from increased reactive oxygen species, neuroinflammation, and neuronal apoptosis in vitro, as well as improving motor deficit, increasing neurotransmitters, and enhancing memory/learning in animal models (in vivo). Given the difficulties involved in the treatment of neurodegenerative diseases, the results presented in this review show promise for the future of clinical research on new therapies for treating these diseases.

Furthermore, these studies with seed extracts open up the possibility of discovering new natural therapies with few side effects compared to those of synthetic drugs. Although the results are promising, the studies are still preliminary, as they are limited to in vitro and in vivo experiments. Therefore, clinical trials are needed in order to extrapolate the results of experimental studies to humans. In addition, clinical trials are essential for determining the safe and effective doses of these seed extracts for treating patients with neurodegenerative diseases.

## Figures and Tables

**Table 1:** Neuroprotective mechanisms of the seeds.

Authorship and Publication Year	Anatomical/Cell Structure AnalyzeD	Experimental Model of Induced Neurodegeneration	Seeds	Extract	Most Relevant Results
Godkar et al. (2003) [26]	Neuronal cultures of rat forebrain	Hydrogen peroxide-induced oxidative damage	*Celastrus paniculatus*	Water soluble extract	Antioxidant
Godkar et al. (2004) [27]	Neuronal cultures of rat forebrain	Glutamate-induced toxicity model	*Celastrus paniculatus*	Water soluble extract	Attenuated toxicity and cell death
Hwang et al. (2004) [28]	Gerbil hippocampus	Neuronal injury induced by transient forebrain ischemia	*Vitis vinifera* L.	-	Protection from ischemic damage
Feng et al. (2007) [29]	Rats	Hypoxic-ischemic brain injury model	*Vitis vinifera* L.	-	Neuronal injury protection
Fujishita et al. (2009) [30]	Primary cultures of astrocytes in the hippocampus	Hydrogen peroxide-induced oxidative damage	*Vitis vinifera*	Polyphenols	Anti-apoptotic and antioxidant
Kasture et al. (2009) [31]	Rats	Parkinson’s disease induced by 1-methyl-4-phenyl-1,2,3,6-tetrahydropyridine	*Mucuna pruriens*	-	No significant antagonism on dopamine neuron degeneration and glial cell activity
Ju et al. (2010) [32]	Rat mesencephalic Pheochromocytoma-12 cells	Model of Parkinson’s disease induced by 6-hydroxydopamine	*Cassia obtusifolia* L.	Ethanolic extract with standardized concentration of rubrofusarin 6- O-gentiobioside	Antioxidant
Jamarkattel-Pandit et al. (2010) [33]	Rat’s brain	Ischemia-reperfusion injury model induced by occlusion of middle cerebral artery	*Sesamum indicum* L.	Defatted seed extract	Reduced the cerebral infarction volume and improved the sensorimotor function
Li et al. (2011) [34]	Neurons in the CA1 region of the rat’s hippocampus	Transient cerebral ischemia due to occlusion of bilateral carotid arteries	*Alpinia katsumadai*	Ethanolic extract	Protection from ischemic damage by neurotrophic factors
Bhuiyan et al. (2012) [35]	Primary cortical neurons of rats	Ischemia model by cell death induced by glutamate and oxygen-glucose deprivation	*Glycine max* L.	Crude ethanol extract of anthocyanins with three isolated components: delphindin-3-glucoside, petuidin-3-glucoside and C3G	Antioxidant and protection against neuronal cell death
Zhao et al. (2012) [36]	Primary cortical neurons of rats	Increased reactive oxygen species and hydrogen peroxide-induced cytotoxicity	*Perilla frutescens* L.	Luteolin isolated extract (BA-kuchiol, 1,3-hydroxybakuchiol, e 3,2-hydroxybaku-chiol)	Antioxidant
Ahmad et al. (2012) [37]	Striated body removed from mice	Model of Parkinson’s disease by 6-hydroxydopamine -induced neurotoxicity	*Sesamum indicum* L.	Oil extraction	Anti-inflammatory, antioxidant, and prevention of neuronal cell death
Sarkaki et al. (2013) [38]	Carotid arteries of female tats	Brain ischemia model induced by the bilateral permanent occlusion of carotid arteries	*Punica granatum*	Ethanolic extract	Antioxidant, significant improvement in passive and active memory and learning impairments
Li et al. (2013) [39]	CA1 region of the gerbil hippocampus	Ischemic damage model induced by transient cerebral ischemia	*Alpinia katsumadai*	-	Antioxidant
Yadav et al. (2013) [40]	Mice	Parkinson’s disease model by chronic exposure to paraquat	*Mucuna pruriens*	Aqueous extract	Antioxidant and improvement of neurobehavioral activity
Ye et al. (2014) [19]	Brain striatum of rats	1-methyl-4-phenyl-1,2,3,6-tetrahydropyridine-induced Parkinson’s disease model	*Camellia oleifera*	Defatted sapogenin extract	Anti-neuroinflammatory and protection of dopaminergic neurons
Xin et al. (2014) [22]	Pheochromocytoma-12 cells	Corticosterone damage	*Alpinia katsumadai*	(2R, 3S) -pinobanksin-3-cinnamate isolated from ethanolic extract	Antioxidant
Noor et al. (2015) [41]	Wistar rats	Animal model of multiple sclerosis with demyelination induced by experimental autoimmune encephalomyelitis	*Nigella sativa*	Oil extraction	Anti-inflammatory and demyelinating
Bhagya et al. (2016) [42]	Rats	Chronic stress-induced cognitive impairment model	*Celastrus paniculatus*	Seed extract oil	Anxiety reduction, improvement in cognitive deficits, restoration of spatial learning and memory
El-Tarras et al. (2016) [43]	Albine rats	Cadmium chloride neurotoxicity model	*Vitis vinifera* L.	-	Decrease in histopathological changes in brain tissue
Yi et al. (2016) [44]	Hippocampus of mice	β amyloid-induced synaptic dysfunction model of Alzheimer’s disease	*Cassia obtusifolia*	Ethanolic extract	Anti-inflammatory effect and significant improvement in memory impairment
Malik et al. (2017) [45]	Cortex and striatum of the rat brain	Huntington’s disease-like symptom model of 3-nitropropionic acid-induced neuronal damage	*Celastrus paniculatus*	Ethanolic extract	Antioxidant
Dehghanian et al. (2017) [16]	Neurons of rat hippocampal subfield CA1	β-amyloid-induced neurodegeneration model of Alzheimer’s disease	*Phoenix dactylifera* L.	Phenolic compounds from methanolic extract	Antioxidant; memory restoration and learning; protection against deterioration of neuronal morphology of the CA1 area of the hippocampus
Shama et al. (2017) [46]	CA1 region of the rat hippocampus	Neurodegeneration and hypoxia-induced oxidative stress	*Cicer microphyllum*	Crude ethanolic extract of compounds (biochanin A, formononetin, genistin)	Antioxidant, reduction of dendritic atrophy in CA1 neurons, significant memory improvement
Wang et al. (2017) [17]	Pheochromocytoma-12 cells neural cells of the rat adrenal medulla	Neuronal apoptotic model with β amyloid 25–35-induced Alzheimer’s Disease	*Litchi chinensis*	Ethanolic extract of saponins	Significant inhibition of apoptosis
Rai et al. (2017) [47]	Mice	Parkinson’s disease model with neuroinflammation induced by 1-methyl-4-phenyl-1,2,3,6-tetrahydropyridine intoxication	*Mucuna pruriens*	Aqueous extract	Anti-inflammatory, antioxidant, and anti-apoptotic
Kong et al. (2017) [48]	Mice	Cerebral injury by ischemia due to the transient occlusion of the middle cerebral artery	*Vitis vinifera* L.	Procyanidin extract	Antioxidant and anti-apoptotic neurotrophic effect
Wang et al. (2017) [49]	Pheochromocytoma-12 cells	Alzheimer’s disease model induced by neuronal apoptosis using β amyloid 25–35	*Litchi chinensis*	Saponin extracts	Anti-apoptotic
Assad et al. (2018) [50]	Rats	Amnesia induced by scopolamine	*Trigonella-foenum graecum* L.	Methanolic extract	Antiamnesic, improvement in the learning and memory process
Luo et al. (2018) [51]	PC-12 neuroblastoma cells	Proteomic evaluation through a neurodegeneration model of oxidative stress induced by hydrogen peroxide	*Vitis vinifera* L.	Procyanidin extract	Antioxidant
Zhou et al. (2018) [52]	Mice’s hippocampus	Cognitive impairment induced by scopolamine injection	*Moringa oleifera*	Ethanolic extract	Antiamnesic, significant improvement in cognitive impairment, increased reactivity of the cholinergic system, hippocampal neurogenesis
Zhou et al. (2018) [21]	Mice	Memory dysfunction and neuroinflammation model induced by lipopolysaccharide	*Cannabis sativa* L.	Hemp seed extracts containing phenylpropionamides	Improved learning and alleviated damage to spatial memory
Fu et al. (2019) [53]	Neuroblastoma N2a cells of mice	Model of ischemic injury by oxygen-glucose deprivation/reoxygenation	*Vitis vinifera* L.	Procyanidin extract	Increased cell viability and anti-apoptotic activity
Wang et al. (2019) [54]	Murine microglia (BV2)	Neuroinflammation induced by lipopolysaccharide	*Cannabis sativa* L.	Ethanolic extract of coumaroylaminobutanol glucopyranoside	Anti-inflammatory and antioxidant
Kim et al. (2019) [18]	Mice’s Pheochromocytoma-12 cells	Alzheimer’s disease model by cytotoxicity induced by hydrogen peroxide with behavioral and cognitive deficit induced by beta amyloid 1–42	*Camellia sinensis*	Oil extract	Antioxidant and significant improvement in cognitive and behavioral dysfunction; protection of cholinergic function
Shalgum et al. (2019) [55]	SH-SY5Y neuroblastoma cells	Toxicicity induced by hydrogen peroxide	*Hibiscus sabdariffa* L.	Ethanolic extract	Antioxidant, lipid peroxidation activity, blocked mitochondrial dysfunction and apoptosis
Song et al. (2019) [56]	HT22 cells from the hippocampus	HT22 cell death by glutamate-induced excitotoxicity	*Reynoutria elliptica*	Isolated methanol extract of procyanidin B2 3″—O-gallate	Antioxidant, inhibition of kinase phosphorylation, protection against apoptotic death and toxicity
Tu et al. (2019) [57]	Murines	Neonatal hypoxic-ischemic brain injury model	*Vitis vinifera* L.	Procyanidin extract	Anti-apoptotic
Wang et al. (2019) [20]	BV2 microglial cells	Model of inflammatory response and lipopolysaccharide-induced oxidative stress	*Cannabis sativa* L.	Cannabisin F extract	Anti-inflammatory and antioxidant
Zeng et al. (2019) [58]	Rats	Ischemic stroke model by acute and late-stage brain damage	*Moringa oleífera*	-	Cerebrovascular protection
Jin et al. (2020) [59]	Primary cultures of hippocampal neurons	Ethanol-induced neuronal damage associated with oxidative stress	*Vitis vinifera* L.	Procyanidin ethanolic extract	Antioxidant
Qiu et al. (2020) [60]	BV-2 and Pheochromocytoma-12 cells	Alzheimer’s disease model of neuroinflammation induced by β amyloid (1–42)	*Litchi chinensis*	Seed polyphenols	Anti-neuroinflammatory and antiapoptotic
Sun et al. (2020) [61]	Rat’s hippocampus	Alzheimer’s disease model of hippocampus neuronal injury induced by β amyloid 25–35	*Litchi chinensis*	-	Injury relief and improvement of cognitive functions via AKT/GSK-3β
Khan et al. (2020) [62]	Neuroblastoma cells (SweAPP N2a) of mice	Alzheimer’s disease model	*Camellia sinensis*	Saponin E1 extract	Reduction of β amyloid
He et al. (2021) [63]	Pheochromocytoma-12 cells	Neuronal damage induced by hydrogen peroxide	*Vitis vinifera* L.	Proanthocyanidin extract	Redução de reactive oxygen species intracelular e inibição de apoptose
Guo et al. (2021) [64]	NSC-34 cell line	Neuronal damage induced by tert-butyl hydro-peroxide	*Celosia argentea* L.	Ethanolic extract from nine saponins and two phenylacetonitrile glycosides	Decrease in reactive oxygen species and cell apoptosis
Rachesee et al. (2021) [65]	BV2 mouse microglial cells	Neuroinflammation induced by lipopolysaccharide	*Mucuna pruriens* (L.) *DC.*	Ethanolic extract	Suppression of inflammatory responses by inhibiting the nuclear factor kappa B signaling pathway

## Data Availability

Not applicable.
